# Classification and Biomarker Genes Selection for Cancer Gene Expression Data Using Random Forest

**Published:** 2017-10-01

**Authors:** Malihe Ram, Ali Najafi, Mohammad Taghi Shakeri

**Affiliations:** 1 *Dept. of Biostatistics, Public Health School, Mashhad University of Medical Sciences, Mashhad, Iran*; 2 *Molecular Biology Research Center, * *Baqiyatallah University of Medical Sciences* *, Tehran, Iran*

**Keywords:** Microarray, Random Forest, Cancer, Gene Selection, Classification

## Abstract

**Background & objective:**

Microarray and next generation sequencing (NGS) data are the important sources to find helpful molecular patterns. Also, the great number of gene expression data increases the challenge of how to identify the biomarkers associated with cancer. The random forest (RF) is used to effectively analyze the problems of large-p and small-n. Therefore, RF can be used to select and rank the genes for the diagnosis and effective treatment of cancer.

**Methods:**

The microarray gene expression data of colon, leukemia, and prostate cancers were collected from public databases. Primary preprocessing was done on them using limma package, and then, the RF classification method was implemented on datasets separately in R software. Finally, the selected genes in each of the cancers were evaluated and compared with those of previous experimental studies and their functionalities were assessed in molecular cancer processes.

**Result:**

The RF method extracted very small sets of genes while it retained its predictive performance. About colon cancer data set *DIEXF*, *GUCA2A*, *CA7*, and *IGHA1* key genes with the accuracy of 87.39 and precision of 85.45 were selected. The *SNCA*, *USP20*, and *SNRPA1* genes were selected for prostate cancer with the accuracy of 73.33 and precision of 66.67. Also, key genes of leukemia data set were *BAG4*, *ANKHD1*-*EIF4EBP3*, *PLXNC1*, and *PCDH9* genes, and the accuracy and precision were 100 and 95.24, respectively.

**Conclusion:**

The current study results showed most of the selected genes involved in the processes and cancerous pathways were previously reported and had an important role in shifting from normal cell to abnormal.

## Introduction

Gene expression profiling, using high-throughput technology in different cells and tissue, is an important source to discover helpful molecular patterns (1). These patterns indicated the genes activity in a cell, and subsequently, the associated states. New technologies such as microarray and next generation sequencing (NGS) are capable of measuring all the genes in a cell or tissue quantitatively every time the experiment is repeated, while providing a large amount of data (2). The great number of gene expression data increases the challenge of how to identify biomarkers and small sets of critical genes associated with disease and the challenge of being able to detect the disease class (3, 4). 

Microarray technology allows studying whole genome, transcriptome, and proteome in different cells as well as tissue and diverse conditions. The yield of this method is very high and it is able to analyze a significant amount of information at a short time. This technique provides the understanding of cellular processes and networks for genome-wide expression profiles (3). Also, gene expression at various time points is a dynamic process, which can be both transiently and permanently changed, and thus, returns different behaviors at the cell and tissue level (1, 4). Classification of complex diseases and cancer based on gene expression profiles is one of the goals of microarray experiment. Traditional classification methods used morphological symptoms and pathological examination that might exist in different types of cancer, and have similar morphological symptoms and pathological patterns. Therefore, it cannot be correctly classified. But, since each disease, including tumor, is basically a poor performance of genes, usage of gene expression data can develop direct detection methods (5).

At the same time that the wide statistical methods influence the medical field, many supervised clustering analysis, and machine learning techniques are developed to deal with gene expression profile data to classify different kinds of diseases or derivatives from the same illness. Various statistical and machine learning methods including clustering (6), the Bayesian algorithm (7), and support vector machine (8) to analyze microarray data are obtained from experiments with high throughput product. Two problems are important in the analysis of gene expression data; first, the high-dimensional genetic and environmental factors in a complex network that are collective and sometimes have multiplicative interaction and make various changes in the gene expression, and the other one is the genetic heterogeneity. Therefore, it is important to use statistical methods to classify such data due to their higher accuracy. On the other hand, as the statistical methods such as regression models have limited ability to deal with genetic heterogeneity, the tree-based non-parametric statistical method is the most powerful, but yet simplest method among the non-parametric statistics methods, and is useful to classify the response variable. Data mining methods are the stronger methods for the real data because the data usually do not give much information; therefore, they could work better and have had better performance for large data. A general rule in most statistical procedures is to determine the distribution of data, which usually prevails the assumption that distribution is normal and finally the correctness or falsity of the final results depends on the correctness of the basic hypothesis (data distribution). But, in the machine learning methods, no assumptions are used about the data, and it makes the differences between these 2 methods. However, as the statistical methods are mathematic-based, they give more exact results than data mining. Statistical methods deal with smaller data sets and when they are applied to large data sets, they increase the probability of error. Since these data have a high noise, the statistical methods are usually employed to remove noise, which increases the computational error itself. Hence, according to the above mentioned reasons, the tree-based statistical methods were used for microarray data analysis. The random forest (RF) method is a modern form of tree-based methods, which was first introduced by Leo Breiman in 2001(6,7). The current study aimed at investigating the performance and the accuracy of RF in gene selection from cancer microarray data that have high prevalence in Iran.

**Random Forest**

In the past the RF algorithm, as an applicable tool, evolved to a standard data analysis tool in high-throughput data.

The RF method is useful in molecular biology studies as it is a simple, interpretive, and flexible method, which can be used for a large number of predictor variables; it is also applicable in limited sample sizes and genetic heterogeneity (7). The most important characteristics of RF are their high potency in determining the role of each variable used in the response prediction (6). Also, it shows an excellent performance even when most predictive variables have noise, the number of variables is much larger than the number of observations, and in problems involving more than 2 classes (8). The RF provides a unique combination of prediction accuracy and model interpretability among popular machine learning methods. The random sampling method and ensemble strategies utilized in RF enable it to achieve accurate predictions as well as better generalizations (9).

Construction of prediction rule for supervised learning problems and ranking variables with respect to their ability to predict the response are the 2 main classes of problems addressed using RF method. The latter is done by considering the so-called variable importance measures (VIMs) and each predictor within the RF algorithm is computed automatically (9).

The following items describe the main features of RF algorithm that make it ideal for the classification of microarray data sets:

1) It is suitable for matrix when there are much more variables than observations. 

2) It is suitable for the 2- and multi-class problems. 

3) When most of the predictive variables are noisy, it has good predictive performance, and therefore, it does not require a pre-selection of genes(10). 

4) Does not over fit. 

5) It can handle a mixture of categorical and continuous predictors and incorporates interactions among predictor variables. 

6) The output is invariant to monotone transformations of the predictors.

7) There are high quality and free implementations: the original Fortran code from L. Breiman and A. Cutler, and an R package from A. Liaw and M. Wiener(11). 

8) Returns measures of important variable (gene).

9) There is little need to fine-tune parameters to achieve excellent performance. The most important parameter to choose is mtry, the number of input variables tried at each split, but it is reported that the default value is often a good choice (11). In addition, the user needs to decide how many trees to grow for each forest (n tree) as well as the minimum size of the terminal nodes (node size).

**Figure1:**
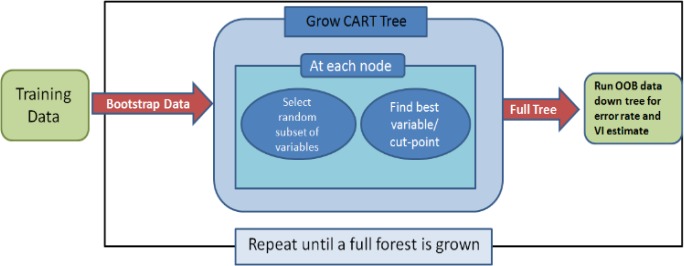
A flowchart for random forest algorithm that represents a workflow for classification problems

Given these promising features, in spite of other prediction methods, it is important to understand the performance of RF with microarray data. For this purpose, the RF algorithms (Figure 1) for both regression and classification explain:

1. Draw n-tree bootstrap sample from the original data.

2. For each of the bootstrap samples, grow an unpruned classification or regression tree, with the following modifications:

a. At each node, rather than choosing the best split among all predictors, randomly sample mtry of the predictors and select the best split from that variable, and based on the splitting separate this node into the 2 nodes.

b. Recursive algorithm partitions continue until the trees reach their largest size (ie, for each observation, a final node) and the node size reaches its smallest value n_min_, without which the tree is pruned.

3. Repeat steps 1 and 2 to make an RF (11, 12).

4. Predict new data by aggregating the predictions of the n-tree trees (ie, majority vote for classification, average for regression).

5. Compute an out-of-bag (OOB) error rate using the data, not in the bootstrap sample.

As observed, RF used both bagging methods (aggregate bootstrap) that successful approach combined unstable learners and variable selection to make the tree.

Common choices for T is 1000 trees and for mtry is √p and log (p) (13), and the minimum node size is 1(14). But in practice, the best value for this parameter depends on the problem and should be treated as a tuning parameter.

RF is too great to interpret; therefore, the quantitative indicators are used to summarize its information. One of these indicators is the important variables. The important variables index is used to rank the variables according to their importance in influencing the response. Variable importance may be caused by interaction with other variables; the most famous important indicators are the Gini importance and permutation importance index(11).

## Materials and Methods

Optimized RF was used to classify cancer, followed by biomarker gene selection for 3 types of cancer. Gene expression raw data used in the current study were retrieved from Gene Expression Omnibus (GEO) and Array Express databases as main, public, and comprehensive databases in deposition of microarray data. Three types of common cancers including colon cancer, prostate cancer, and leukemia were selected and the raw data were downloaded. These types of cancer have high prevalence and mortality rates in the developing countries such as Iran. 

The current study used 3 microarray data sets as follows:

Leukemia (GSE9476): This dataset contained 64 samples and 22 283 genes that data were divided into 2 groups of 38 control and 26 cancer samples. These data were recorded in GEO database on 01 July, 2016 (15).

Prostate cancer (GSE71783): This data set consisted of 30 samples and 17 881 genes that data were divided into 2 groups of 15 control and 15 cancer samples. These data were recorded in GEO database on 30 October, 2007 (16).

Colon cancer (GSE44861): This data set included 111 samples and 22 277 genes that data were divided into 2 groups of 55 control and 56 cancer samples. These data were recorded in GEO database on 05 March, 2013 (17).

**Table 1:** The Number of Genes and Samples Used in the Current Study for the Considered Cancers

Class	Sample (+/–)	Gene	Data Set
No tumor/tumor	111(56/55)	22277	**Colon cancer**
Normal/tumor	30(15/15)	17881	**Prostate cancer**
Normal/leukemia	64(26/38)	22283	**Leukemia**

All the preprocessing, processing, and statistical analyses were performed using R software version 3.3.1. Limma (the linear models for microarray data) package is an R software package used to analyze gene expression data obtained by microarray techniques and/or RNA sequencing.Limma is used to analyze the designed experiments and the assessment of differential expression by the linear models. The empirical Bayesian methods are used to provide stable results even when the number of arrays is small. The linear model and differential expression functions are applied to all microarray technologies. And also Limma package is used to analyze and preprocess the species of microarray platforms (18, 19). In the current study, the eBayes function was used to extract significant differential gene expression (a P-value of <0.05 was considered statistically significant); after processing, the significant genes were introduced in a table.

**Table 2:** Evaluation Criteria of Random Forest Classifier Model

Kappa	Precision	Accuracy	Specificity	Sensitivity	Overall Error	
**74.8**	85.45455	87.38739	85.45455	89.28571	12.61261	**Colon cancer**
**46.7**	66.66667	73.33333	66.66667	80	26.66667	**Prostate cancer**
**90.2**	100	95.2381	100	88.46154	4.761905	**Leukemia**

**Figure 2:**
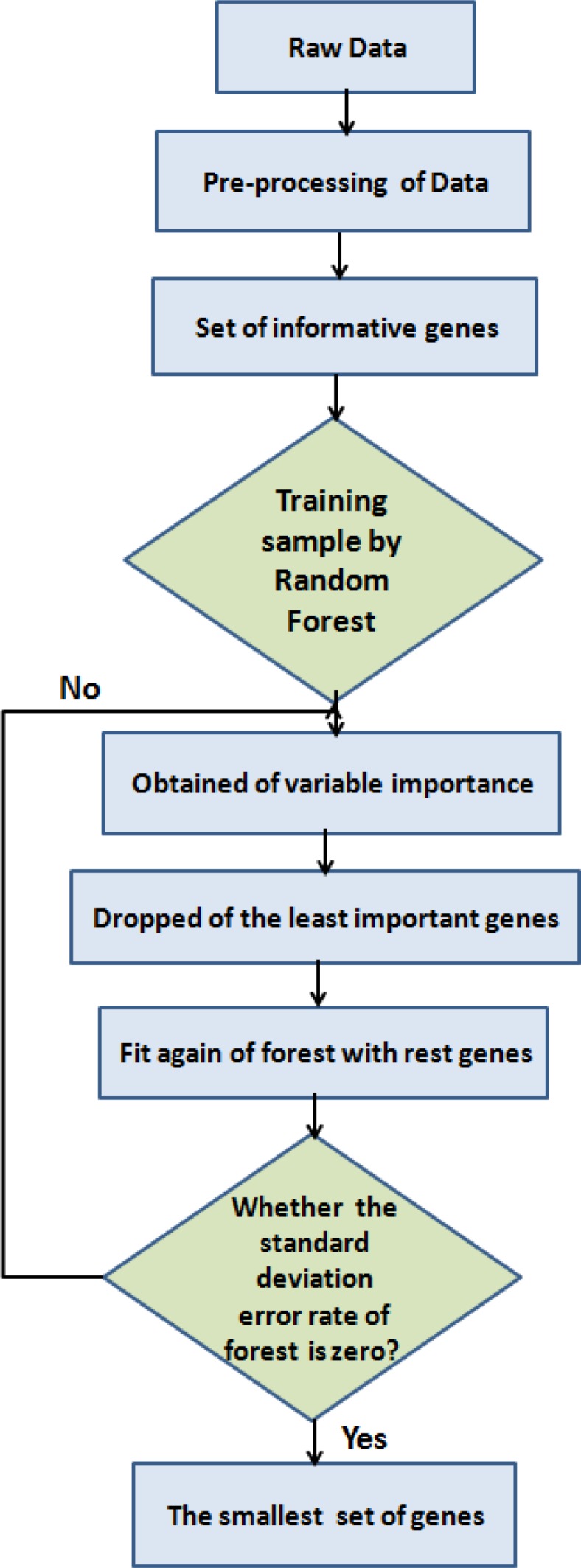
The process steps of feature selection and classification of microarray data that used in this study using Random Forest package

After processing the data and the extracted informative genes, data were trained by RF method. The importance of each gene was calculated; then, they were ranked and the smallest set of gene was extracted. All these steps were performed by the Random Forest package that is schematically described in Figure 2.

To obtain the smallest set of genes, the iterative random forest was fitted at each iteration step for building a new forest through discarding the lowest variable importance. The selected set of genes is the set that yields the smallest OOB error rate. Then, all rest forests (the least important genes) are iteratively tested. By default, fraction.dropped = 0.2, which allows 20% of the genes in each stage, that is at the bottom of the ranking list of the variables is removed and the new forest fits again, while 20% of genes are dropped. Variable importance is not recalculated at each step because severe overfitting results from recalculating variable importance. The solution with the smallest number of genes is selected; its error rate is within the U standard errors of the minimum error rate of all forests. The process to remove the least important genes and fit again the forest continues until the minimum standard deviation (SD) of all forest error rates are zero. The smallest set of genes is the same set that its minimum SD of error of the entire forest is zero (20). This step was performed with the varSelRF software package.

## Results

Table 1 introduces 3 cancer data sets and shows the number of primary genes and samples used in the study. After primary preprocessing on all data sets, 2000 differential expressed genes (DEGs) were extracted and the analysis was performed with this number of genes. The RF is a classifier model fitted on all 3 data sets with metrics (assessment criterions) (Table 2). 

EGFR, epidermal growth factor receptor; VHL, the von Hippel-Lindau disease; HIF, hypoxia-inducible factor; SNRPA1, small nuclear ribonucleoprotein polypeptide A'; GO, gene ontology TNF-R1, tumor necrosis factor receptor type; DR, death receptor 

Tables 3, 4, and 5 show the smallest gene set for different data sets selected by RF. These genes are known as cancer biomarkers and the function of each of these genes is presented in the mentioned tables. The function and annotation of each selected gene was extracted from valid databases such as NCBI, Uniprot, and Genecard.

According to the results in Table 2, RF could classify cancer data sets. IGHA1, immunoglobulin heavy constant alpha 1

**Table 3:** The Smallest Set of Genes Selected From Colon Cancer Data by the Random Forest Method

Function	Gene. Symbol	Probe ID
Regulates the p53 pathway to control the expansion growth of digestive organs	DIEXF	**"X204700_x_at"**
Guanyin, cyclase C binding protein 2A, increasing intracellular cGMP. Endogenous activator of intestinal guanylate cyclase. It stimulates this enzyme through the same receptor binding region as the heat-stable enterotoxins.	GUCA2A	**"X207003_at"**
Carbonic anhydrases are a large family of zinc metalloenzymes that catalyze the reversible hydration of carbon dioxide. They participate in a variety of biological processes, including respiration, calcification, acid-base balance, bone resorption, and the formation of aqueous humor, cerebrospinal fluid, saliva, and gastric acid.	CA7	**"X207504_at"**
IGHA1 is a Protein Coding gene. Among its related pathways are Vesicle-mediated transport and Regulation of nuclear SMAD2/3 signaling.	IGHA1	**"X215118_s_at"**

**Table 4:** The Smallest Set of Genes Selected From Prostate Cancer Data by the Random Forest Method

Function	Gene. Symbol			Probe ID	
Alpha-synuclein is a member of the synuclein family, which also includes beta- and gamma-synuclein. Among its related pathways are “transport to the Golgi” and subsequent modification and “EGFR1 signaling pathway”.		SNCA	**X2777714**	
This gene encodes an ubiquitin specific processing protease that was first identified as a substrate of the VHL protein E3 ubiquitin ligase complex. In addition to being ubiquitinated by the VHL-E3 ligase complex, this enzyme deubiquitinates HIF-1 alpha, and thereby, causes increased expression of HIF-1alpha-targeted genes, which play a role in angiogenesis, glucose metabolism, cell proliferation, and metastasis.		USP20	**X3191176**	
SNRPA is a protein coding gene. Among its related pathways are gene expression and mRNA splicing-major pathway. GO annotations related to this gene include *poly (A) RNA binding*.		SNRPA1	**X3642162**	

**Table 5:** The Smallest Set of Genes Selected From Leukemia Cancer Data by the Random Forest Method

Function	Gene. Symbol	Probe ID
The ANKHD1-EIF4EBP3 mRNA is an infrequent, but naturally occurring read through transcript of the neighboring ANKHD1 and EIF4EBP3 genes. This read through transcript encodes a protein composed mostly of the multiple ankyrin repeats, single KH-domain protein, with its C-terminus encoded in a different reading frame from the shared portion of the EIF4EBP3 gene.	ANKHD1-EIF4EBP3	**208773_s_at**
This gene encodes a member of the plexin family. Plexins are transmembrane receptors for semaphorins, a large family of proteins that regulate axon guidance, cell motility and migration, and the immune response. The encoded protein and its ligand regulate melanocyte adhesion, and viral semaphorins may modulate the immune response by binding to this receptor. The encoded protein may be a tumor suppressor for melanoma.	PLXNC1	**213241_at**	
The protein encoded by this gene is a member of the BAG1-related protein family. BAG1 is an anti-apoptotic protein that functions through interactions with a variety of cell apoptosis and growth related proteins including BCL-2, Raf-protein kinase, steroid hormone receptors, growth factor receptors, and members of the heat shock protein 70-kDa family. This protein was associated with the death domain of TNF-R1 and DR3, and thereby negatively regulates downstream cell death signaling. The regulatory role of this protein in cell death was demonstrated in epithelial cells that undergo apoptosis while integrin mediated matrix contacts are lost.	BAG4	**219624_at**
This gene encodes a member of the protocadherin family, and cadherin superfamily of transmembrane proteins containing cadherin domains. These proteins mediate cell adhesion in neural tissue in the presence of calcium. The encoded protein may be involved in signaling at neuronal synaptic junctions. Sharing a characteristic with other protocadherin genes, this gene has a notably large exon that encodes multiple cadherin domains and a transmembrane region.	PCDH9	**219737_s_at**	

## Discussion

Microarray data are highly asymmetric and have a lot of noise. In addition, expression data represent the number of genes, which usually reaches thousands of genes, much more than the number of samples, that can dramatically increase the computational cost of data processing (21). However, from a biological point of view only a small subset of genes are related to disease and as key genes play a role in the disease, and more genes are often inappropriate and have noise that could negatively affect the performance of classification system. Therefore, choosing at least a minimal gene list is one of the most important applications in the analysis of microarray data, which are effective in complex diseases. The target of gene selection is a selection of a subset of useful and appropriate genes to diagnose the disease among the whole genes that leads to improve the accuracy and speed of the classification system (22).

Zhang et al., conducted a comparative study of feature selection and multiclass classification, and used a 4-fold cross validation instead of bootstrap to assess the rate of errors. Their approach that pre-selects a set of 150 genes for prediction used a set of 7 different algorithms and 8 different rank selection methods. But, in their research, no algorithm or gene selection algorithm was consistently the best. The method used in the study, with 2 classification algorithms and gene selection methods, showed that RF classification and selection gene had the best performance (23).

Jiang et al., conducted a joint analysis of 2 microarray gene expression data sets to select lung adenocarcinoma marker genes that recompute variable importance at each step and their gene selection is based on both the OOB error and the prediction error. They also showed the excellent performance of variable selection using the RF for their data sets (24).

A heuristic strategy of variable selection using RF was developed by Genuer et al. (13). They used basic workflow of varSelRF and ranked all features by *VIMP*. In this study, the unimportant variables were removed by setting a threshold for the minimum prediction value from CART fitting. The applied procedure kept m important variables and then implemented the nested RF, starting from the most important variable and increasing the number of variables in a stepwise fashion until all m variables were transferred. The final model was selected on the basis of OOB error.

Diaz-Uriarte et al., to return variable selection in each iteration, made a RF and in each iteration, part of the predictors were removed by the least important variable and selected subset of the remaining predictors. Then, selected a subset-optimal that had the smallest frequency error or the least area under the curve(22, 25).

In the present study, first, a tree named "primary" with all features was produced. Then, in each repetition, one of the features was removed. Therefore, the average amount of error rate diversity was calculated for each feature based on the recently-created-tree and the primary one. Thus, the precision obtained in the last step, a higher weight with better precision, was allocated. Then, deletion of variables from random forest using OOB standard as minimal standard was done by successive deletion of least important variables until the standard deviation of the least error rate of all the forest got zero and the smallest genomic set was obtained and at last several key genes were selected for each cancer.

## Conclusion

Selected genes were assessed biologically from their revenue in biological processes and pathways. Results showed that most of the selected genes were involved in processes and pathways, which had an important role in cancer progression. In other words, these genes had the main role in the progress and dysregulation of a biological process to induce cancer state in the cell. Apoptosis is a process that these genes act in; if this process faces a disorder, the cell gets immortalized or cancerous. For example, *DIEXF* gene (in the list of colon cancer) and *BAG4 *gene (in the list of leukemia) act in the apoptosis process. The angiogenesis is another process getting disorder in cancerous states (*PLXNC1* and *PCDH9* genes in the list of leukemia). The process of contact and intracellular connections and cellular migration (*PLXNC1* and *PCDH9* genes in leukemia) and other processes such as activating EGFR and SMAD pathways were the other processes that selected genes in this project were involved to control and regulate the processes.

## Conflict of interest statement

The authors declared no conflict of interest.
